# Optimization of electroporation method and promoter evaluation for type-1 methanotroph, *Methylotuvimicrobium alcaliphilum*

**DOI:** 10.3389/fbioe.2024.1412410

**Published:** 2024-05-15

**Authors:** Shubhasish Goswami, Steven W. Singer, Blake A. Simmons, Deepika Awasthi

**Affiliations:** ^1^ Biological Systems and Engineering Division, Lawrence Berkeley National Laboratory, Berkeley, CA, United States; ^2^ Joint BioEnergy Institute, Emeryville, CA, United States

**Keywords:** electroporation, *Methylomicrobium alcaliphilum*, constitutive promoter, inducible promoter, methanotrophs

## Abstract

Methanotrophic bacteria are promising hosts for methane bioconversion to biochemicals or bioproducts. However, due to limitations associated with long genetic manipulation timelines and, lack of choice in genetic tools required for strain engineering, methanotrophs are currently not employed for bioconversion technologies. In this study, a rapid and reproducible electroporation protocol is developed for type 1 methanotroph, *Methylotuvimicrobium alcaliphilum* using common laboratory solutions, analyzing optimal electroshock voltages and post-shock cell recovery time. Successful reproducibility of the developed method was achieved when different replicative plasmids were assessed on lab adapted vs. wild-type *M. alcaliphilum* strains (DASS vs. DSM19304). Overall, a ∼ 3-fold decrease in time is reported with use of electroporation protocol developed here, compared to conjugation, which is the traditionally employed approach. Additionally, an inducible (3-methyl benzoate) and a constitutive (sucrose phosphate synthase) promoter is characterized for their strength in driving gene expression.

## Introduction

Methane (CH_4_) is one of the most potent greenhouse gases (GHGs), as 1 mol of CH_4_ can absorb about 24 times more radiations than 1 mol of carbon dioxide (Wuebbles and Hayhoe, 2002). In the past decades, rising anthropogenic activities like biomass burning, biological waste, landfills (renewables) and extraction of natural gas, coal and petroleum (non-renewables) have contributed more to global atmospheric methane emissions than natural sources (Wuebbles and Hayhoe, 2002; Strong et al., 2015). Methane also adversely impacts climate by the slow but steady oxidation of CH_4_ to CO_2_ in the atmosphere and the rapid conversion to CO_2_ due to burning of natural gas as a fuel source (Boucher et al., 2009). The cost of natural gas, which is ∼90% methane, has been consistently lower than sugar prices, making it a low-cost feedstock for bioconversion to biochemicals, biofuels and bioproducts. Biological conversion of methane to other value-added materials has the potential to provide a sustainable, eco-friendly route to chemical production (Hwang et al., 2018). Moreover, bioconversion of methane has a higher carbon conversion efficiency compared to its chemical conversion, where oxidation of methane to methanol using methanotrophs has 75% conversion efficiency versus 20%–50% using chemical processes (In Yeub et al., 2014; In Yeub et al., 2015). With recent advancements in synthetic biology, methanotrophs have been used for successful production of variety of biochemicals and demonstration of proof-of-concept studies with highly selective methane bioconversion routes to many specialty chemicals as well. A recent review has discussed a list of biochemicals that can be produced such as polyhydroxyalkanoates, methanol, single cell protein, ectoine, fatty acids, lipids and organic acids (Gęsicka et al., 2021), and 90% utilization of gaseous substrate including methane for isobutanol production (Liang et al., 2022) using methanotrophs as host organisms.

Methanotrophs, or microbes that consume CH_4_, provide a biological and natural methane sink either via aerobic or anaerobic routes of CH_4_ consumption (Rhee et al., 2019). However, among the methanotrophs, aerobic methanotrophic bacteria have been mostly isolated in pure cultures and studied (Guerrero-Cruz et al., 2021). There are two types of aerobic methanotrophs, type-I methanotrophs (gammaproteobacterial) and type-II methanotrophs (alphaproteobacterial) (Hanson and Hanson, 1996). Amongst the two, type-I methanotrophs employ a more energy efficient pathway for formaldehyde assimilation into cellular biomass, using the ribulose monophosphate pathway coupled with the Entner-Doudoroff or Emden Myerhoff Parnas pathways. Therefore, type-I methanotrophs could be attractive candidates to develop as a microbial platform for biochemical/fuel production (Hanson and Hanson, 1996). Consequently, type-I methanotrophs belonging to the Gram-negative genus *Methylotuvimicrobium*, such as *M. buryatense* and *M. alcaliphilum*, have been previously engineered to produce fatty acid (Demidenko et al., 2017), 2,3-butanediol (Nguyen et al., 2018), lactic acid (Henard et al., 2016), and rhamnolipids (Awasthi et al., 2022). However, unlike sugar based model industrial microbial hosts such as, *Escherichia coli* and *Bacillus subtilis*, methanotrophic hosts have very limited tools for genetic manipulation (Henard and Guarnieri, 2018), hindering efforts at metabolic engineering. Therefore, advancement of genetic tools, such as faster transformation methods and characterized promoters for gene expression, is imperative to expand the utility of *Methylomicrobium* sp. as future microbial cell factories.

Conjugation-based transformation methods have been used conventionally for plasmid transfer in methanotrophs (Ojala et al., 2011; Puri et al., 2015). However, the challenges of conjugation for cloning or expression purposes include the involvement of multiple, time-consuming steps and that the recipient strain is susceptible to genetic changes that may occur in the plasmid during conjugation (Puri et al., 2015). Replacing conjugation with electroporation provides advantages by shortening the cloning process and providing a direct mode of DNA transfer. Recently, electroporation transformation was reported in type-I methanotrophs *M. buryatense* 5G; however, transformed colonies via electroporation were only obtained when plasmids were first conjugated into *M. buryatense* 5G, isolated from this strain, and then re-transformed to *M. buryatense*, as no colonies were obtained after electroporation when plasmids were directly isolated from *E. coli* strain TOP10 (Yan et al., 2016). Thus, this method also was indirect and required additional steps. Hence, in this study, a robust electroporation protocol was developed after evaluation and optimization of four parameters effecting electroporation efficiency. In our understanding, this is the first study to show comprehensive analysis of electroporation parameter evaluation for a methanotrophic bacteria and present a working/reproducible electroporation protocol.

Further on, metabolic engineering often requires heterologous gene expression, and so a variety of promoters are needed to enable robust strain engineering. A few constitutive and inducible promoters have been previously used by different groups in genus *Methylotuvimicrobium* (Puri et al., 2015; Henard and Guarnieri, 2018). Therefore, in addition to improved transformation methods, more characterized promoters would be beneficial for the advancement of genetic tools. In this study, an inducible (3-methyl benzoate) and a constitutive (sucrose phosphate synthase) promoter were screened with green-fluorescent protein (GFP) reporter tag to monitor their gene expression strengths in *M. alcaliphilum* strain DASS, previously developed by our group as a high fatty acid producer (Awasthi et al., 2022). Robust Protocols developed for strain *M. alcaliphilum* strain DASS are expected to enhance the application of this strain as a potential industrial microbe for bioconverting CH_4_ to targeted bioproducts, with implications for tool development in other type-I and/or type-II methanotrophic bacteria.

## Materials and methods

### Bacterial growth and culture conditions

All bacterial strains and plasmids used in this study are listed in Table 1. *E. coli* was used as an intermediate host for plasmid cloning and transformation. *E. coli* strains were grown in Luria-Bertani (LB) media and 50 μg/mL kanamycin was added to the culture when required. *E. coli* was cultivated at 37°C, shaking at 200 RPM. *M. alcaliphilum* strains were cultured as described previously by Awasthi et al. (2022). In brief, *M. alcaliphilum* was grown in sealed serum bottles at 30°C at 200 RPM in Pi (π)/P3 media with 30% (w/v) NaCl, 100 μg/mL Kanamycin (kan) was added to the growth medium when required. Cells were incubated under CH_4_ (Ultra-pure 99.9%, Airgas) and air at ratio 1:1. *M. alcaliphilum* cell growth (OD_600_) was measured by spectrophotometer SpectraMax M2 microplate reader (Molecular devices, San Jose, CA, United States). Colony selection after transformation was performed in P_3_ media-agar plates kept in the anaerobic jar (Oxoid, Remel) under CH_4_- air (1:1) atmosphere for 5–6 days.

**TABLE 1:** List of bacterial strains and plasmids used in the study.

Name	Description	References
Plasmids		
pCAH01	Expression vector, P_tetA_ *bla-tetR* CoE1ori F1 *oriV, oriT, traJ, trfA, ahp.* (7.6 kb; IncP origin of replication)	Henard et al. (2016)
pCAH01_emGFP	pCHAO1 with P_tet__emGFP (8.3 kb; IncP origin of replication)	Henard et al. (2016)
pCM433	Genome integration vector for gene knock outs or inserts, with kanR, *traJ*, *oriT* (5.3 kb; shorter version of IncP origin of replication)	Puri et al. (2015)
pAWP78	Cloning vector with kanR, *oriV*, *oriT*, *traJ*, *trfA* (4.9 kb; IncP origin of replication)	Puri et al. (2015)
pCM184	Cloning vector, Ap^r,^ Kn^r^, Tc^r^; *traJ*, *OriT*, (6.7 kb; IncP origin of replication)	Marx and Lidstrom (2002)
pDA21	pCAH01 with Psps-promoter (5.71 kb)	Awasthi et al. (2022)
pSGDA1	pCAH01 with Pm-promoter, #138475 (pORTMAGE-Pa1) purchased form Addgene (10.2 kb)	JPUB_022465
pSGDA2	pCAH01 with Psps-emGFP (9.1 kb)	JPUB_022467
Strains		
*E. coli* TOP10F	F[*lac*I^q^ *Tn10(tet* ^*R*^ *)] mcrA Δ(mrr-hsdRMS-mcrBC) φ80lacZΔM15 ΔlacX74 deoR nupG recA1 araD139 Δ(ara-leu)7697 galU galK rpsL(Str* ^*R*^ *) endA1 λ* ^-^	Invitrogen
*E. coli* DH5α	F^–^ *endA1* *glnV44* *thi1* *recA1* *relA1* *gyrA96* *deoR* *nupG* *purB20* φ80d*lacZ*ΔM15 Δ(*lacZYA-argF*) U169, hsdR17(*r*_*K*_^–^ *m*_*K*_^+^), λ^–^	Invitrogen
*E. coli* NEB Express^®^ (#C2523)	*fhu*A2 [lon] *ompT gal sulA11* R(*mcr*-73::miniTn10—*Tet* ^S^)2 [*dcm*] R(zgb-210::Tn10—*Tet* ^S^) *endA1* Δ(*mcrC-mrr*)114::IS10	NEB
*E. coli* GM272	F-, *fhuA2* or *fhuA31*, *lacY1* or *lacZ4, tsx-1* or *tsx-78*, *glnX44*(AS), *galK2*(Oc), λ-, *dcm-*6, *dam*-3, *mtlA*2, *metB*1, *thiE,1 hsdS21*	JPUB_022468
*M. alcaliphilum* DSM19304	Wild type	Awasthi et al. (2022)
*M. alcaliphilum* DASS	Strain DSM19304 adapted for tolerance to rhamnolipids, fatty acid producer	Awasthi et al. (2022)

### Electroporation optimization

For plasmid electroporation, a single colony of strain DASS was inoculated in 10 mL P3 media in 50 mL serum bottle incubated at 30°C with 200 RPM shaking. The culture was grown until an OD_600_ of 1.4 was reached. This culture was used as the primary inoculum, and 500 µL was subsequently added to final growth culture, 50 mL P_0.75_ low salt (7.5% (wt/vol) NaCl) media in a 180 mL serum bottle followed by incubation at 30°C with shaking at 200 RPM until OD_600_ 0.8. A liquid: gas ratio of 1:5 was used for the seed culture as has been used previously (Awasthi et al., 2022), which is in the commonly used liquid: gas ratio for aerobic methanotrophs (Henard et al., 2019). For the secondary culture an alternate liquid: gas ratio of 1:4 was used (50 mL Pi media in 180 mL serum bottle) to obtain enough biomass at OD_600_ 0.8. Cells were harvested at 5,000 RPM at 4°C for 15 min by centrifugation (Avanti J-15R, Beckman Coulter, IN, United States). Cell pellet obtained was then resuspended in 50 mL of electroporation wash buffer (as mentioned). All the wash buffers were kept at 4°C during experimental procedures. Washing was repeated three times at 5,000 RPM, 4°C for 15 min with the wash buffer. After the third wash, final cell pellet was resuspended in 150 μL of wash buffer. 60 μL of cells were transferred to electroporation cuvette (1 mm gap). Gene Pulser XCell™ (BioRad Labs GmbH, Munich, Germany) was used for electroporation. Plasmid concentration was kept at 1 μg for each electroporation transformation based on a previous report in related studies (Yan et al., 2016). Additionally, in this work a lower concentration (around 500–600 ng) of pDNA was also tested that resulted in very low/no transformation efficiency. Hence, for all electroporation experiments a standard pDNA concentration of 1 µg was used. Various electroporation voltages (as mentioned) ranging from 1.4 to 2.2 kV were screened to identify the optimum voltage. Immediately after electroporation, cells from cuvette were transferred to 10 mL of P3 media in 50 mL serum bottle, followed by recovery at 30°C under methane: air atmosphere for the duration as mentioned. After recovery the growing cells were harvested at 5,000 RPM at room temperature for 15 min, followed by plating on P3 (kanamycin) selection plates and incubated at 30°C in anaerobic jar under CH_4_: air (1:1) atmosphere. Colonies were observed and tabulated in 5–6 days after incubation.

### Plasmid construction

All plasmid construction was performed using NEB HiFi assembly conceptually based on Gibson assembly (Gibson et al., 2009) using *E. coli* TOP10F as the initial plasmid propagating host. The primers used in this study are listed in Table 2. Inducible promoter, P_m_ (3-methyl benzoate) was cloned from plasmid 138475 (pORTMAGE-Pa1) purchased form Addgene. The P_m_ promoter (1.946 kb) fragment was PCR amplified using primers P_m_ fragment forward/reverse (Table 2) and the vector pCAH01_emGFP was linearized by PCR amplification using primers pCAH01_emGFP vector forward/reverse (Table 2), the PCR amplicons of promoter and linearized vector were assembled using NEB HiFi assembly kit, resulting in pSGDA1. Similarly, sucrose phosphate synthase promoter (P_sps_; 792 bp) was PCR amplified using primers P_sps_ fragment forward/reverse (Table 2) with template pDA21 (Table 1), vector pCAH01_emGFP was linearized using primers P_sps_ vector forward/reverse (Table 2). Vector and insert were assembled using HiFi assembly (NEB) to construct pSGDA2.

**TABLE 2:** List of primers used in this study.

List of primers	Primer’s sequence (5′to 3′)
pCAH01_emGFP vector forward	acc​act​ccc​tat​cag​tga​tag​ag
pCAH01_emGFP vector reverse	caa​aaa​tta​gga​att​aat​cat​ctg​gcc
P_sps_ vector forward	cct​aat​ttt​tgg​gta​ctc​aaa​aag​ccg​gtc​gtg
P_sps_ vector reverse	gga​gtg​gca​cga​aca​act​atc​tca​agt​gac​gct
P_sps_ fragment forward	tgt​tcg​tgc​cac​tcc​cta​tca​gtg​ata​gag​aaa​ag
P_sps_ fragment reverse	ttt​ttg​agt​acc​caa​aaa​tta​gga​att​aat​cat​ctg​gcc​att​cga​t
P_m_ fragment forward	cta​att​ttt​gtc​aag​cca​ctt​cct​ttt​tgc​att​gac
P_m_ fragment reverse	gggagtggtttgcataaagcctaaggggtaggn
P_m_ vector forward	ttt​atg​caa​acc​act​ccc​tat​cag​tga​tag​aga​aaa​g
P_m_ vector reverse	aag​tgg​ctt​gac​aaa​aat​tag​gaa​tta​atc​atc​tgg​cca​t

## Results

### Optimization of electroporation for *M. alcaliphilum*

**FIGURE 1:**
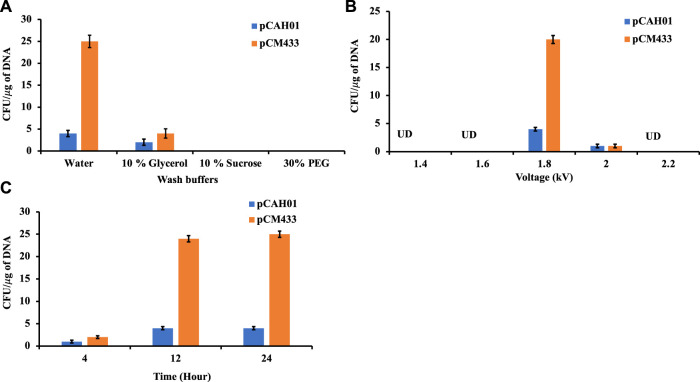
Evaluation of different electroporation conditions for plasmid transformation in *Methylotuvimicrobium alcaliphilum* strain DASS. **(A)** Effect of wash buffers on electroporation efficiency; **(B)** Impact of voltage range; **(C)** Post electroporation cell recovery time. UD, undetected.

Conjugation is the traditional mode of plasmid (pDNA) transformation for methanotrophs which takes about 4–5 weeks. In this work, electroporation-based transformation method was evaluated and optimized to accelerate the pDNA transfer procedure. Two plasmids, pCAH01 (7.6 kb size; expression vector; Table1) and pCM433 (5.3 kb size; cloning vector; Table 1) were initially evaluated to identify the optimal conditions of a) wash buffers, b) voltage, and c) cell recovery time. Both plasmids were first isolated from *E. coli* TOP10F and subsequently electroporated to *M. alcaliphilum* DASS. Four wash buffers (autoclaved Milli Q water, 10% (w/v) sucrose, 10% (v/v) glycerol, 30% (v/v) polyethylene glycol (PEG) that have been previously shown to work in either *M. buryatense*, *E. coli*, *Bacillus* or *Staphylococcus carnosus* (Okamoto et al., 1997; Löfblom et al., 2007; Wu et al., 2010; Yan et al., 2016) were evaluated to enhance electroporation efficiency for *M. alcaliphilum*. A standard voltage of 1.8 kV was used for this experiment, as has been reported earlier (Elmore et al., 2023). Transformation efficiency was calculated based on the number of colony forming units (CFUs)/µg of plasmid DNA. As shown in Figure 1A, no CFUs were observed on plates with cells washed with 10% sucrose and 30% PEG, indicating that these solutions were not effective under the conditions tested in this transformation process. Plasmids pCAH01 and pCM433 yielded 3 and 4 CFU/μg DNA, respectively, when 10% glycerol was used as wash buffer. The highest CFUs were reported with the use of autoclaved milli Q water, resulting in ∼25 CFU/μg of plasmid for pCM433 (Figure 1A).

Next, the effect of different electroporation voltages (1.4, 1.6, 1.8, 2.0, 2.2 kV) was tested. CFUs were observed only on 1.8 and 2.0 kV treated cells, with ∼20 fold for pCM433 and ∼4-fold for pCAH01 more CFUs at 1.8 kV than 2.0 kV (Figure 1B). Post shock cell recovery time is a vital factor for viability of cells exposed to extreme voltage shocks. Thus, the duration of cell recovery time can impact the transformation efficiency as observed in the experiments. To evaluate this for *M. alcaliphilum* DASS, cells were cultivated for 4, 12, and 24 h post electroporation in P3 medium under methane: air (1:1) atmosphere (Figure 1C). Both 12 and 24 h recovery times had comparable CFUs/µg DNA, which were 3- fold higher than CFUs observed for the 4 h recovery, indicating that the post-shock cell recovery time impacts transformation efficiency, and 12 h incubation is optimal. Overall, the optimized electroporation conditions established by these experiments include three critical parameters-cell wash by autoclaved milliQ water, electroporation at 1.8 kV and 12 h post shock cell recovery time for strain *M. alcaliphilum* DASS.

**FIGURE 2:**
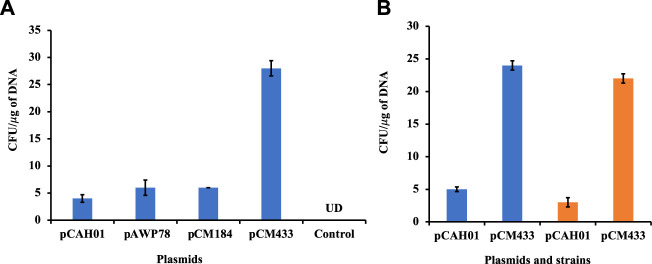
Evaluation of electroporation protocol reproducibility in *M. alcaliphilum*. **(A)** Using different replicative vectors; **(B)** In different variants of *M. alcaliphilum*, strains DASS (blue) and wild-type DSM19304 (orange). UD, undetected.

The optimized condition was then assessed on other replicative plasmids pAWP78 and pCM184 (plasmids of varying sizes; Table 1) for *M. alcaliphilum* (Figure 2A). The pDNA transformation was successful in each trial (*n* = 4), confirming the replication of protocol is irrespective of sizes and, cloning or expression plasmid (Table 1). *M. alcaliphilum* DASS is an evolved strain (Awasthi et al., 2022), and resulting mutations could have impacted the transformation efficiency relative to the parent strain. Therefore, the optimized electroporation protocol was evaluated for the wild type (WT) parent strain DSM19304 to establish the reproducibility of the developed method. Data (Figure 2B) shows the transformation procedure was reproducible in WT.

In the scope of improving transformation efficiency of plasmids, another strategy that employs specific methylation-based plasmid propagation was also evaluated. Bacterial endonucleases differentiate native DNA from foreign DNA due to differential DNA methylation at specific, short (∼4–8 nucleotides) sequences with using a DNA methyl transferase to protect the host chromosome at sites that are cleaved by a corresponding restriction enzyme. Thus, unmethylated or improperly methylated foreign DNA will be degraded upon entering the cell (Vasu and Nagaraja, 2013). In *E. coli*, two type of and site-specific DNA methyl transferases- Dam and Dcm have been reported, encoded by *dam* (DNA adenine methyltransferase) and *dcm* (DNA cytosine methyltransferase), respectively (Marinus and Løbner-Olesen, 2014). Notably, it has been mentioned that Dam and Dcm methylation pattern might decrease the transformation efficiency in alternate hosts (Marinus and Løbner-Olesen, 2014). Therefore, to further optimize the pDNA transformation efficiency, prior to electroporation in *M. alcaliphilum*, pCAH01 was propagated in various *E. coli* strains (Table 1): TOP10F (*dam* ^*+*^ *;* DNA adenine methyltransferase, *dcm* ^*+*^; DNA cytosine methyltransferase), C2523 (*Δdcm*), GM272 (*Δdam Δdcm*). No colonies were obtained on plates with transformed plasmids isolated from *E. coli* strains C2523, GM272 (Supplementary Figure S1). Similar results after the repeated trials (*n* = 3) depicts that *dam* and *dcm* methylation of *E. coli* enables transformation of plasmid in *M. alcaliphilum*, and absence of any methylation pattern were deleterious. In future studies, *Methylomicrobium* specific methylation should be evaluated if it enhances plasmid transformation efficiency as has been reported in *E. coli* strain AG5645 (Riley et al., 2023).

### Promoter evaluation for heterologous gene expression

**FIGURE 3:**
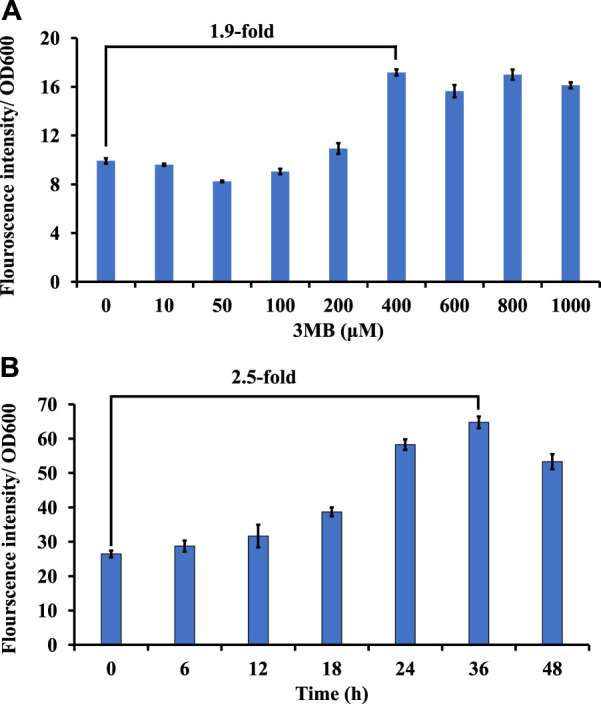
Screening of inducible and constitutive promoters using GFP as a reporter in strain DASS. **(A)** Growth-normalized fluorescence intensity (GFP_409_/OD_600_) for P_m_ promoter in response to 3MB concentration. **(B)** Growth-normalized fluorescence intensity (GFP_409_/OD_600_) for P_sps_ promoter in P3 media.

Promoters driving gene expression are critical for rational genetic engineering. However, the availability of well characterized promoter systems for inducible and/or constitutive gene expression in non-model hosts like *M. alcaliphilum* is limited compared to model organisms like *E. coli*. In this work, to create another inducible system, the 3-Methylbenzoate (MB) inducible promoter (P_m_) of *Pseudomonas putida* (Marqués et al., 1999) was evaluated in *M. alcaliphilum* using green florescent protein as a reporter on plasmid pSGDA1 (Table 1; Figure 3A). *M. alcaliphilum* (pSGDA1) cultures were induced by addition of 3MB in increasing concentrations up to 48 h, and 3MB dose dependent response on growth and fluorescence was monitored. Growth of host, measured as optical density (OD_600nm_), was mostly unaffected by increasing concentration of 3MB till 400 μM, monitored until 48 h (Supplementary Figure S2A). The normalized GFP fluorescence intensity was observed to have increased almost 2-fold from 100 to 400 μM of 3MB, followed by intensity saturation by further increase in 3MB concentrations, indicating optimal activity of P_m_ promoter in presence of 400 μM 3MB (Figure 3A).

Constitutive promoters are known for their constant expression of genes in a host cell, across different set of culturing conditions (Li et al., 2015). This host constitutively produces considerate amount of sucrose to maintain osmotic balance in its native halo-alkaline environment (But et al., 2015). Thus, recently, the constitutive sucrose phosphate synthase promoter (P_sps_) was shown to drive expression of a recombinant rhamnolipid pathway in strain DASS (Awasthi et al., 2022); however, the strength of this promoter remained uncharacterized. To study the strength and duration of expression, GFP was expressed under the P_sps_ (pSGDA2; Figure 3B). Strain DASS (pSGDA2) cultures were grown till 48 h (Supplementary Figure S2B). Gene expression was observed to be coupled to cell growth, and it peaked at 24–36 h, at the early to mid-stationary phase of growth (Figure 3B) with 2.5-fold increase compared to 0 h.

## Discussion

**FIGURE 4:**
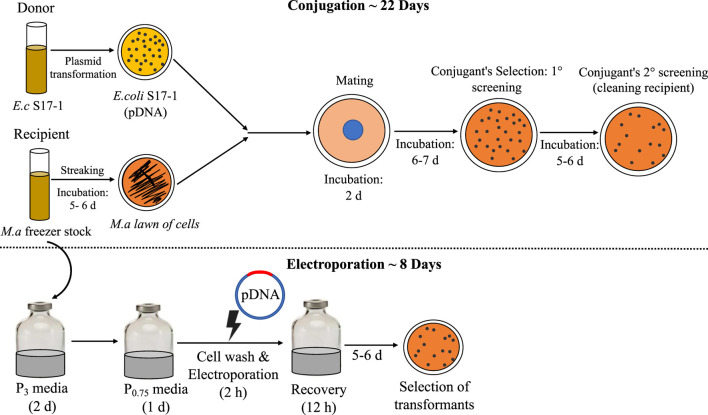
Comparison of conjugation and electroporation: the steps involved and timeline. *E.c*, *E. coli*; *M.a*, *M. alcaliphilum*.

Conjugation is the traditional mode of plasmid (pDNA) transformation for methanotrophs, which requires multiple steps and a long time (∼4 weeks) (Puri et al., 2015), a process limitation that unfortunately holds back the host from fast genetic manipulation time-line, a trait sought for microbial biocatalysts. Thus, in the efforts to make *M. alcaliphilum* a robust host for strain engineering, it is imperative to address the limitations of established pDNA transformation methods. Electroporation is a one-step, clean pDNA transfer procedure, which also prevents mutations that may occur during plasmid transfer to multiple hosts for mating or conjugating (Huang and Wilks, 2017). Thus, direct plasmid electroporation is time saving, also when compared to other methods demonstrated in methanotrophs, where the re-transformation of plasmids is required from methanotrophs strain back into cloning strain (Yan et al., 2016). In this work, the highest CFUs were reported with the use of autoclaved milli Q water, resulting in ∼25 CFU/μg of plasmid for pCM433. This result affirms the use of autoclaved milli Q water as has been previously shown to work for electroporation in *M. buryatense* 5G (Yan et al., 2016). The efficiency of transformation represented by CFU/μg DNA, was consistently higher for pCM433 in all trials. pCM433 plasmid is 2.3 kb lesser in size than pCHA01 (Table 1). It was reported previously that transformation efficiency decreases linearly with increase in plasmid size (Hanahan, 1983). This may explain the lesser obtained CFU/μg DNA in pCHA01 compared to pCM433, in this study. Finally, the resulting workflow (Figure 4) shows the overall impact of improving this method, resulting in ∼3 times reduction in the duration and effort of implementing electroporation for heterologous DNA transfer in *M. alcaliphilum* for expression and cloning vectors. Currently, industrial organisms such as *Escherichia coli*, *Saccharomyces cerevisiae*, *Pichia pastoris* take around 12 h, 2–6 days and 4–6 days respectively to show colonies in selection plates after plasmids transformation. Here in *M*. *alcaliphilum*, with the presented approach, colony appearance occurs at ∼5–6 days after transformation in antibiotic selections plates. Therefore, electroporation may help bring the methanotrophs closer to other commonly used industrial hosts in DNA transfer and colony selection timeline.

Regulated promoter-gene systems are vital for gene expression during strain engineering to control/tune recombinant product formation. Thus, to add to the promoter library in *M. alcaliphilum,* strength of a constitutive promoter P_sps_ (sucrose phosphate synthase) and an inducible promoter, P_m_ (3-methyl benzoate) were evaluated. In this work, P_m_ promoter showed a 2-fold increase in presence of 400 µM 3MB. Very recently, the P_m_ promoter was shown to be 5.7-fold in presence of 1 mM benzoate in *Methylococcus capsulatus* (Jeong et al., 2023). Though, concentration of inducer above ≥0.4 mM were found to be adversely impacting the growth of the host (Supplementary Figure S2A), the results of this study establish the robustness of the P_m_ promoter across the genus. A small decrease in OD of P_m_ promoter by 0.2–0.3 in magnitude, at 36–48 h was observed only at higher concentrations (800–1,000 μM) of 3MB (Supplementary Figure S2B) indicating a stress response by cell at high dose of inducer concentrations (Shis and Bennett, 2013). As an obligate methanotroph, *M. alcaliphilum* is incapable to metabolize aromatic substrates as a growth or energy source therefore, aromatic substrate-based inducers are a potential range of promoters that could be further evaluated for this host.

Using a constitutive promoter for a desired rate of heterologous gene expression and biochemical production is a common practice in strain engineering (Bienick et al., 2014; Yan and Fong, 2017). In this direction, constitutive gene expression has been previously studied in the *Methylotuvimicrobium* genus under promoters expressing housekeeping genes like, *pmo*C, *pmo*A, *pmo*B (particulate methane monooxygenase), *mxa* (methanol dehydrogenase) and *hpi* (hexulose phosphate isomerase) (Henard and Guarnieri, 2018). A recent report showcased the strength of constitutive promoters such as P_DnaA_ (2.81-fold), P_Integrase_ (3.45-fold), P_rpmB_ (22.09-fold), P_(2Fe–2S)-binding protein_ (41.54-fold) using GFP as reporter protein *in Methylomonas* sp. DH-1 (Lee et al., 2021). P_sps_ earlier studied by our group (Awasthi et al., 2022) is shown to efficiently perform 2.5-fold increase in gene expression, in this work. Thus, the endogenous promoter- P_sps,_ inducible promoter- P_m_, and electroporation protocol developed in this study, aids to the advancement of genetic engineering practices in *M. alcaliphilum* and should be evaluated and expanded to other type-1 methanotrophs.

## Conclusion

The type-I methanotroph *M. alcaliphilum* is capable of methane bioconversion and has the potential to become a model cell factory to produce biochemicals and bioproducts from methane. Advancements in genetic tools for these microbes are needed to accelerate their use as-a model host to study methanotrophy, and/or an industrial host. This study showcased an optimized and reproducible electroporation-based plasmid transformation protocol that reduces the time of plasmid transformation from 4–5 weeks to 2 weeks as compared to conventional conjugation-based method. The materials employed for the developed electroporation method are standard materials and an economical wash solution-autoclaved milli Q water. The reproducibility of this protocol was shown with different cloning and expression vectors, as well as, in the lab adapted strain DASS and the wild type parent strain, DSM19304. Additionally, a responsive inducible promoter P_m_ (inducer 3MB) and a growth based constitutive promoter (P_sps_) were established for driving efficient gene expression. These methods and promoter systems can be further evaluated for their effectiveness and reproducibility in other type-I methanotrophs.

## Data Availability

The raw data supporting the conclusion of this article will be made available by the authors, without undue reservation.
