# Supplementary material for: Optimization of electroporation method and promoter evaluation for type-1 methanotroph, Methylotuvimicrobium alcaliphilum
Source: Front Bioeng Biotechnol. 2024 May 15;12:1412410. doi: 10.3389/fbioe.2024.1412410 (PMC11133525; doi:10.3389/fbioe.2024.1412410)
Supplement: Supplementary file 1 [file DataSheet1.docx]

**Optimization of Electroporation method and Promoter Screening for type-1 methanotroph, *Methylotuvimicrobium alcaliphilum***

Shubhasish Goswami^1^, Steven W. Singer^1,2^, Blake A. Simmons^1,2^ and Deepika Awasthi^1,2*^

^1^Biological Systems and Engineering Division, Lawrence Berkeley National Laboratory, Berkeley, CA- 94720

^2^Joint BioEnergy Institute, Emeryville, CA-94608

*Corresponding Author

Dr. Deepika Awasthi

Biological Systems and Engineering Division

Lawrence Berkeley National Laboratory

Berkeley, CA 94720 (U.S.A.)

Email: [dawasthi@lbl.gov](mailto:dawasthi@lbl.gov)

Figure S1: Transformation efficiency of *M. alcaliphilum* with differently methylated pCAH01 isolated from specified *E. coli* strains. UD, undetected.

Figure S2: A) Growth (OD_600_) of strain DASS (pSGDA1) with increasing inducer 3-methyl benzoate (3MB) concentrations. B) Growth (OD_600_) of strain DASS (pSGDA2).
